# Unpacking Value Tensions in Older Adults’ Engagement with Generative AI

**DOI:** 10.1093/geroni/igaf122.4041

**Published:** 2025-12-31

**Authors:** Debaleena Chattopadhyay, Tasneem Mubashshira

**Affiliations:** University of Illinois Chicago, Chicago, Illinois, United States; University of Illinois Chicago, Chicago, Illinois, United States

## Abstract

Generative artificial intelligence (GAI) refers to AI systems that create new content—such as text, images, audio, or video—based on patterns learned from large datasets. Unlike traditional digital technologies that are task-specific and predictable, GAI systems support flexible, open-ended interaction. However, this flexibility introduces several challenges, including unpredictable outputs (e.g., hallucinations), ambiguous authorship, and humanlike interactions, especially in conversational tools. We used a value sensitive design approach and reflexive thematic analysis to examine how community-dwelling older adults engage with GAI. Semi-structured interviews with twelve U.S.-based older adults (median age = 64; median CFI = 2.5) explored their perceptions, experiences, and concerns. Participants (n = 8) who used tools like Copilot or ChatGPT described using them for small, routine tasks such as finding information and editing text (“for the little things”; “find out things that I’ve forgotten”) and appreciated their ease of use (“I could tell it more precisely what I wanted”; “answers me back in detail”; “pretty simple to use”). However, both users and non-users expressed value tensions, particularly around trust (“It’s functional, but I think part of it is I don’t trust it”), lack of accountability (“I mean who’s giving you those answers?”), absence of informed consent (“an excuse to legitimize thievery”), algorithmic bias (“there’s so much misinformation out there”), and broader concerns about societal harm (“it scares me about what human nature will do with AI”). Notably, even frequent users demonstrated critical engagement rather than overtrust, pointing to the need for design practices that engage with—not undermine—users’ healthy skepticism.

